# Chemical and Medical Experiments on the Diabetes Sauar

**Published:** 1803-01-01

**Authors:** C. Nicholas, C. Gueudeville

**Affiliations:** M. N. I. Paris; Paris


*Chemical and Medical Experiments on the Diabetes Sauar, by* C. Nicholas, M. N. I. and C. Gueudeville, M. D. *of Paris.*

THE presence of sugar, which for a long time was supposed to exist only in the sugar cane, is now known to exist not only in the vegetable, but even in the mineral kingdom, as is proved by the experiments of C. Vauquelin on the emerald, &c. Some of the products of milk had shown a matter analogous to sugar; and our countryman, Willis, had long ago remarked, in the affection in question, the sweet taste of the urine. C. Nicholas and Gueudeville, having met in their practice many people attacked with diabetes, instituted a series of comparative experiments on the urine of persons afflicted with diabetes, and that of persons in health. They have proved, by a number of experiments, the existence of the sauara-muscous substance in this first, and from which they extracted the acetous and alcohol, whilst the latter or healthy urine was not found susceptible of any fermentation either spirituous or acetous. This memoir comprehends a history of the supposed causes of this disease, and of the methods of cure from the days of Hipprocrates to the present, together with a description of all the symptoms. The following are the principal points of this memoir: This disease is more frequent in countries where general use is made of cyder or drinks of this kind; it is owing principally *to a continual spasmodic derivation of the nutritious juices unanimalized on the urinary organs, which also changes the gastric, pancreatic, and biliary fluids, &c.!* It appears to be peculiar to muscular temperaments; its seat is in the organs of digestion, and the urinary organs supply by the excess of their evacuations, the other secretions and excretions, which in this case become suspended. The urine, which, as we have already stated, runs into vinous and acetous fermentation, gives an alcohol of a disagreeable smell, a sugar which crystallizes, the nature of which is not as yet well known, instead of the uric and benzoic acids, which it should contain; the ammoniacal and phosphoric salts are in small quantity; the blood of people labouring under this disease is very serous, and contains but little of the above salts. These phenomena offer to the authors the following indications. 1st. To take off spasm. 2dly. *To restore to the nutritious juices the principle of animation!* To accomplish this, we must chuse food and remedies from among those substances which contain azot and phosphoric salts; and after these indications, the authors prescribe a regimen of animal food composed of fat meat, and milk as drink, in which may may be dissolved phosphat of soda. As medicine, they recommend bolusses formed of the watery extract of opium, and wine, and sometimes musk. This treatment has been followed by compleat success.

